# Proactive Vitality Management, Work–Home Enrichment, and Performance: A Two-Wave Cross-Lagged Study on Entrepreneurs

**DOI:** 10.3389/fpsyg.2022.761958

**Published:** 2022-03-03

**Authors:** Luca Tisu, Delia Vîrgă

**Affiliations:** Department of Psychology, West University of Timişoara, Timişoara, Romania

**Keywords:** work–home enrichment, entrepreneurial performance, cross-lagged, mediation, individual strategies, positive psychology, proactive vitality management

## Abstract

This study provides a cross-lagged examination of the relationships between proactive vitality management, work–home enrichment, and entrepreneurial performance. Specifically, based on the Job Demands-Resources and Conservation of Resources theories, we postulate a mediation model where proactive vitality management leads to entrepreneurs transferring resources developed in their work role to thrive in their home role (i.e., work–home enrichment), resulting in augmented entrepreneurial performance. The hypotheses were tested with data collected at two time points, 1 onth apart—T1 (*N* = 277) and T2 (*N* = 249), from Romanian entrepreneurs. We analyzed autoregressive, causal, reversed, and reciprocal models to test the mediation model. In the linkage between predictor and outcome variable, the reversed model is the best-fitting model, showing that proactive vitality management is only a distal precursor of performance. However, the best-fitting models for the relationship between predictor and mediator and between mediator and outcome were the reciprocal models. Thus, proactive vitality management and work–home enrichment have reciprocal effects on each other over time, as was the case between work–home enrichment and entrepreneurial performance. These results are in line with the resource gain cycle perspective of the Conservation of Resources theory. Employing proactive behaviors to optimize functioning at work enables the transfer of resources to the home role. Potentiating one role through aspects of another will thus generate additional resources reflecting on entrepreneurial performance. Hence, this study provides insights into precursors and mechanisms that can shape entrepreneurial performance.

## Introduction

Research on entrepreneurs as individuals uncovered various indicators that entrepreneurs consider when evaluating their business success. For entrepreneurs, personal factors, such as entrepreneurial wellbeing and work–life balance, are vital elements in determining entrepreneurial success, being equal to or even outranking business profitability and income indicators (Walker and Brown, 2004; Gorgievski et al., 2011; Kirkwood, 2016). Indeed, it has been argued that the frustration of personal factors may lead to business exit decisions, even when the financial indicators of the business are satisfactory (Wach et al., 2016). Thus, to safeguard the societal and economic benefits generated by entrepreneurship (e.g., innovation and vacant job creation; Bosma and Kelley, 2019; Shir and Ryff, 2021), it appears crucial to uncover antecedents and mechanisms that allow entrepreneurs to satisfy their personal and business needs.

In this respect, scholars have already identified various fixed (e.g., openness to experience; Franco and Prata, 2019) and malleable (e.g., entrepreneurial self-efficacy; Miao et al., 2017) entrepreneurial characteristics linked to business performance and entrepreneurial wellbeing (for synthesis, see Stephan, 2018). However, two major gaps in knowledge still need to be addressed. First, there is a need to identify behavioral tools to help entrepreneurs thrive both in their work and home roles. Although a certain overlap exists between factors that predict entrepreneurs’ satisfaction with their business and their personal life (e.g., human capital and personality traits), most of these constructs are rather non-developable. As such, Kleine and Schmitt (2021) heed researchers to focus on malleable constructs when investigating entrepreneurs’ wellbeing and success determinants, emphasizing incorporating behavioral components into future research models.

Second, empirical evidence regarding potential positive causal links between entrepreneurs’ work and home roles is scarce. To date, there is consistent proof of a negative effect between the two roles entrepreneurs assume, which detracts from their wellbeing and performance (for synthesis, see Stephan, 2018; Kleine and Schmitt, 2021). Being an entrepreneur and having an active social life beyond this role appears to lead to higher entrepreneurial strain (Arshi et al., 2020, 2021). This is probably why some entrepreneurs tend to sacrifice their personal life to fully commit to establishing and managing their business (Ezzedeen and Zikic, 2017; Godin et al., 2017; Adisa et al., 2019). Yet, if their role in their private life encumbers some entrepreneurs, why do others consider personal and family-related factors as essential success indicators for their business (Walker and Brown, 2004; Gorgievski et al., 2011; Kirkwood, 2016)? Are they setting impossible standards and aspirations, or can the work and home role of entrepreneurs also act as allies? Due to a lack of synchronous investigations that examine positive causal links between the work and home role of entrepreneurs, this question remains, to date, unanswered.

Anchored in positive psychology (Seligman and Csikszentmihalyi, 2014), the present study aims to initiate closing these gaps. Specifically, following the Job Demands-Resources (JD-R; Bakker and Demerouti, 2017) theory, we propose proactive vitality management—moldable behaviors that allow individuals to manage their physical and mental energies to achieve optimal functioning at work (Op den Kamp et al., 2018), as an antecedent of entrepreneurial performance. Entrepreneurial performance refers to entrepreneurs’ satisfaction with business-related outcomes, such as profitability or number of employees (Gorgievski et al., 2014). Entrepreneurs who engage in behaviors that allow them to be vital and energetic at work (e.g., preparing a nutritious breakfast prior to a busy workday) should have the energy to capitalize on existing opportunities, leading to increased entrepreneurial performance. Additionally, based on the Conservation of Resources (COR; Hobfoll, 2011) theory, we test one component of work–home enrichment (Geurts et al., 2005; Greenhaus and Powell, 2006), namely, work-to-home enrichment (hereinafter work–home enrichment), as a mediator between proactive vitality management and entrepreneurial performance. Work–home enrichment encompasses aspects of the work role that, through a spillover effect, enhance functioning in the home role (Geurts et al., 2005; Greenhaus and Powell, 2006). Being able to enrich their home role through aspects of their work should potentiate entrepreneurial performance because entrepreneurs will possess an extended resources reservoir to invest in their business (e.g., social support; Powell and Eddleston, 2013), and perceive that their personal aspirations are being satisfied (Walker and Brown, 2004; Kirkwood, 2016).

Thus, JD-R and COR theories are employed complementary to support the proposed model. On the one hand, COR theory explains how aspects pertaining to entrepreneurs’ work and home roles can be seen as resources that can be employed from one role to potentiate aspects of another (Hobfoll, 2011). For instance, entrepreneurs could make use of their autonomy (work resource) to spend time with friends and family, thus gaining social support (home resource). In turn, experiencing social support will boost entrepreneurs’ wellbeing (Kleine and Schmitt, 2021) and, subsequently, their performance and that of their business (Powell and Eddleston, 2013; Stephan, 2018). However, to gain additional resources an intentional investment of existing resources is necessary (Hobfoll, 2011). To illustrate, imagine someone who wishes to buy a product. Indeed, as a resource, money is needed to acquire said product. Nevertheless, possessing sufficient money does not equivalate with obtaining the product. The action of buying is needed as a catalyst in the process. Without handing over the money to the vendor (intentional behavior), the transaction would not occur, and the product would not be obtained. Similarly, although entrepreneurs may possess the relevant resources (e.g., autonomy and energy) to balance their work and home roles, they may fail to engage in proactive behaviors that can help them invest those resources into both roles in an adequate manner. The JD-R theory proposes such intentional behaviors, known as individual strategies (Demerouti et al., 2019), upon which entrepreneurs can rely to alter their environment and maximize the effect of existing resources (Bakker and Demerouti, 2017). Proactive vitality management is one such individual strategy (Op den Kamp et al., 2018). Thus, by learning to manage their energies optimally at work (i.e., proactive vitality management), entrepreneurs could make use of existing resources (e.g., autonomy) to attract further resources in another role (e.g., social support). Being able to potentiate their home role through aspects of their work role (i.e., work–home enrichment) will reflect, as argued above, in their entrepreneurial performance.

Following another tenet of COR theory, we further posit that the proposed mediation model can capture a positive gain spiral (Hobfoll, 2011) where the variables have mutual positive effects on each other over time. Being satisfied with their business performance should permit entrepreneurs to reinvest their existing resources (e.g., time and energy) in other performance-enhancing behaviors. For instance, they could channel the positive state generated by satisfaction with their business to be more receptive when interacting with friends or family or start jogging in the morning to manage their energy levels further. Following this reasoning, the roles entrepreneurs assume (i.e., work vs. home) could be potential allies, cyclically potentiating each other, allowing entrepreneurs to thrive in, and be satisfied by their personal and work life concomitantly. We test this assumption by employing a two-wave cross-lagged design.

Thus, regarding specific contributions of this investigation, the proposed cross-lagged mediation model seeks to (1) shift the perspective from factors contributing to entrepreneurs experiencing work–home conflict, to factors that can help entrepreneurs thrive in both roles (Stephan, 2018; Kleine and Schmitt, 2021), (2) establish proactive vitality management as an antecedent of work–home enrichment and entrepreneurial performance and, (3) capture a positive longitudinal causal relationship between entrepreneurs’ work and home role. This will provide empirical evidence as to why entrepreneurs should seek to reconcile the two apparently competing roles (Hobfoll, 2011; Ezzedeen and Zikic, 2017; Godin et al., 2017) while also uncovering a moldable behavioral tool that can be employed to achieve this objective (i.e., proactive vitality management). Being able to potentiate the home role through aspects of their work should thus help entrepreneurs be fully satisfied with their chosen career path (Gorgievski et al., 2011; Kirkwood, 2016; Stephan, 2018) and assure the success of their entrepreneurial venture.

### Proactive Vitality Management and Entrepreneurial Performance

The JD-R theory is well known for explaining the interplay between contextual resources (e.g., autonomy and social support) and work performance, employing a motivational component (i.e., work engagement) as an explanatory mechanism (Bakker and Demerouti, 2017). When individuals perceive they have sufficient resources at their disposal, they become more energetic and dedicated in their work, reflecting on their performance. Furthermore, a recent expansion of the JD-R theory led to the inclusion of behavioral self-regulatory strategies that individuals employ to maximize existing resources and avoid loss, known as individual strategies (Demerouti et al., 2019). In short, the new JD-R extension argues that individuals actively seek to capitalize on existing resources to enhance their performance. For instance, individuals can make use of existing growth opportunities (contextual resources) to proactively develop new critical skills that can enable them to handle their work role better (individual strategy), resulting in increased performance (Tisu et al., 2021; Tisu and Vîrgă, 2021). Although initially developed to reflect processes in the wage-employed sector, existing research demonstrates that the JD-R theory can also be successfully used to explain various processes in the entrepreneurship domain as well (Dijkhuizen et al., 2016; Dinh et al., 2021; Kleine and Schmitt, 2021). Considering this, we have selected one individual strategy stemming from the JD-R theory—proactive vitality management—as a precursor of entrepreneurial performance.

Proactive vitality management encompasses “individual, goal-oriented behaviors aimed at managing physical and mental energy to promote optimal functioning at work” (Op den Kamp et al., 2018 p. 493). These behaviors are self-initiated and idiosyncratic, meaning that individuals decide when, where, and how they employ them. Furthermore, they act as catalysts that can direct an investment of existing resources into other resource-generating activities. As past research demonstrates, managing, and directing one’s energies as needed yields an increase in role-prescribed (Op den Kamp et al., 2018; Ye et al., 2020) and creative performance (Bakker et al., 2020; Op den Kamp et al., 2020). Proactive vitality management is also an alternative to recovery activities, ensuring individuals have sufficient energetic resources at their disposal for endeavors they engage in (Op den Kamp et al., 2018). This is particularly important for entrepreneurs, where the uncertainty inherent to their career path can rapidly deplete their resources (Hahn et al., 2012; Laguna et al., 2017), reflecting on lower entrepreneurial performance (Stephan, 2018).

To replenish their resource–reservoir, entrepreneurs could detach from work through recovery activities (e.g., microbreak; Fritz et al., 2011). However, as a recent diary study demonstrates, entrepreneurs find it hard to detach from their job due to work-related stressors (Wach et al., 2021). This impedes their recovery, affecting their immediate wellbeing (Wach et al., 2021), thus reducing their performance (Stephan, 2018). Proactive vitality management represents a viable solution to this problem, potentially securing an enhancement of entrepreneurial performance. As past research indicates, proactivity is, indeed, linked to entrepreneurs’ wellbeing (Hahn et al., 2012) and performance (Rauch et al., 2009). Being able to proactively manage one’s energy, ensuring it remains constant throughout the workday, means entrepreneurs will be equipped with the necessary resources to face challenges that arise in their work roles. Imagine an entrepreneur who must deliver an important presentation to secure additional funds from investors. Should said entrepreneur take a few minutes prior to the presentation to meditate about the meaning of her work and reflect on how the service/product positively impacts the community (i.e., proactive vitality management) is bound to enhance her energy levels. She will have additional resources to draw from during the presentation and be more vibrant and convincing in her speech, thus securing the much-needed funds. By proactively managing her energy levels, she can succeed in attracting supplementary resources that can help her business grow, reflecting in her entrepreneurial performance.

Furthermore, proactive vitality management is a suitable individual strategy, especially for this occupational category, because most prerequisites necessary for employing such proactive behaviors are met in the case of entrepreneurs. To engage in proactive vitality management, individuals must be able to organize their work in a fashion that accommodates the inclusion of such proactive behaviors in their daily routine (i.e., autonomy; Op den Kamp et al., 2018) and be motivated to employ these self-regulatory behaviors (Cangiano et al., 2021). Two hallmarks of entrepreneurship are the autonomy entrepreneurs experience in their work (Stephan, 2018) and their autonomous motivation (Stephan et al., 2020). Thus, entrepreneurs should be motivated to make us of their autonomy to start the day by allotting time for physical exercise prior to beginning the workday. While commuting, they could listen to lively music to energize themselves. Engaging in such self-regulatory actions encompassed by proactive vitality management is bound to increase entrepreneurs’ energy levels, thus generating additional resources that can be invested in their work. For instance, empirical evidence suggests that proactive vitality management is linked to increased creativity (Bakker et al., 2020; Op den Kamp et al., 2020). Thus, entrepreneurs who engage in proactive vitality management could generate and use said resources (e.g., creative thinking) to devise an improvement on current products, thus securing a competitive advantage in the market (Zhou, 2015). This will positively impact how entrepreneurs perceive their business, resulting in enhanced entrepreneurial performance.

Additionally, based on COR theory, reciprocal effects, known as positive gain spirals, are to be expected (Hobfoll, 2011). Those entrepreneurs who are satisfied with their business should also seek to engage in proactive vitality management further. This assumption is in line with the proposition of COR theory which stipulates that those who have more resources at their disposal are also more prone to invest in and secure additional resources (Hobfoll, 2011). For instance, being happy with their business’s income can reduce entrepreneurs’ work-related strain and generate additional resources, such as leisure time. Entrepreneurs can capitalize on this resource to physical exercise in the morning or visit a museum for inspiration. Engaging in proactive vitality management will thus energize them for the day, allowing them, in turn, to perform better at work and increase their entrepreneurial performance. Based on the arguments above, we expect:

*Hypothesis 1a:* Proactive vitality management at T1 has a positive impact on entrepreneurial performance at T2.

*Hypothesis 1b:* Entrepreneurial performance at T1 has a positive impact on proactive vitality management at T2.

*Hypothesis  1c:*  Proactive vitality management and entrepreneurial performance have positive and reciprocal effects on each other over time.

### Proactive Vitality Management and Entrepreneurial Performance: Work–Home Enrichment as a Mediator

The theoretical considerations of COR theory also led us to postulate that the relationship between proactive vitality management and entrepreneurial performance is mediated by entrepreneurs transferring resources developed in their work role to enhance functioning in their home role. According to COR theory, people employ various resources to attract, foster, and protect anything they value and deem important (Hobfoll, 2011). Considering that entrepreneurs deem their personal and business life to be equally important (Walker and Brown, 2004; Kirkwood, 2016), they should be motivated to invest existing resources in both roles. Thus, from a COR perspective, engaging in self-regulatory strategies (e.g., proactive vitality management) can be seen as a behavioral resource investment process (e.g., energy and time) that generates resources in the work role (e.g., increased creative performance; Bakker et al., 2020). According to Greenhaus and Powell (2006), both the positive behavior that led to the accumulation of resources (e.g., museum visit) and the consequential positive state generated by it (e.g., positive affect) can then be transferred to the home role, enhancing entrepreneurs’ functioning in their role as individuals. This process is known as work–home enrichment (Geurts et al., 2005; Greenhaus and Powell, 2006).

**Figure 1:**
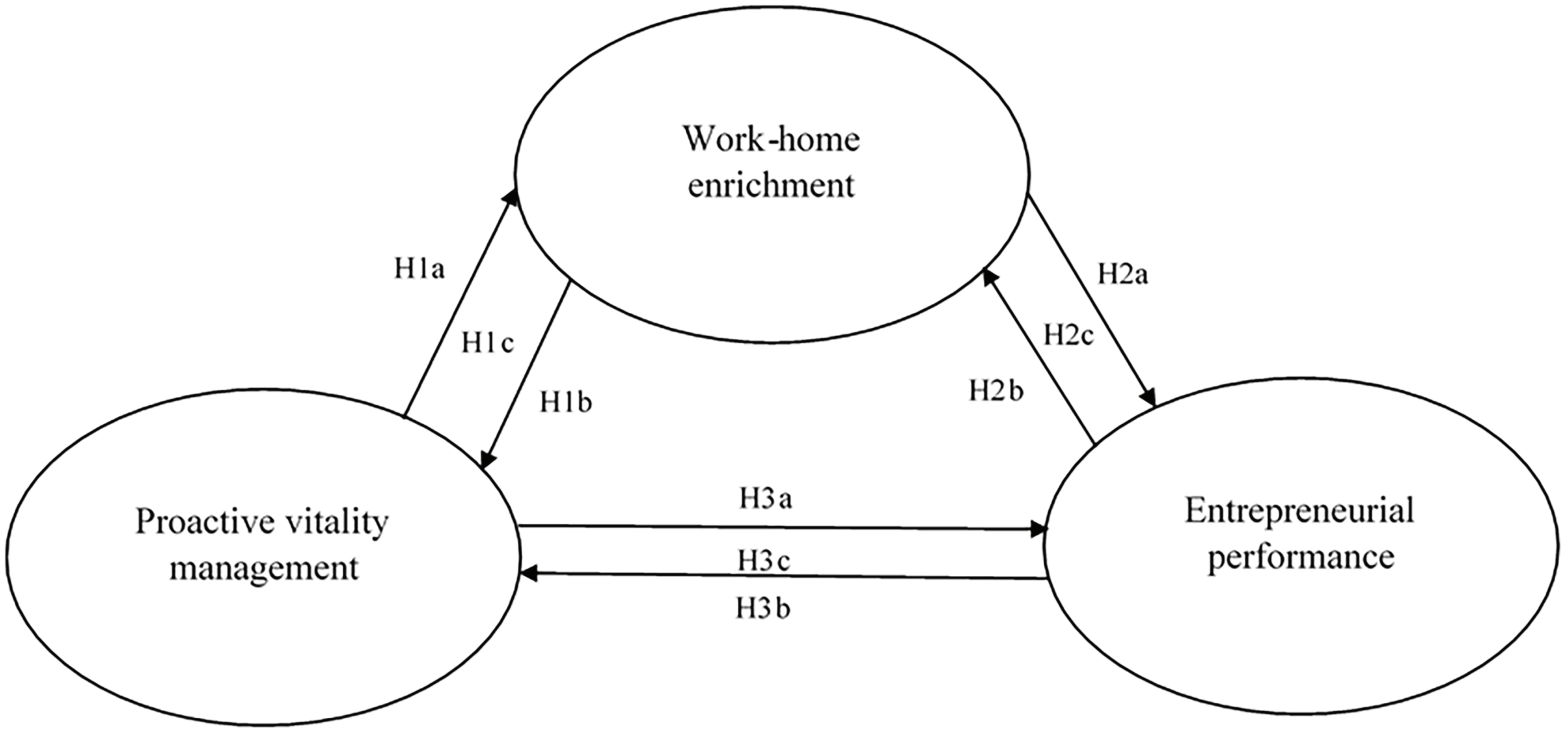
The hypothesized mediation model.

Work–home enrichment is one of the four components of work–home interaction (Geurts et al., 2005; Greenhaus and Powell, 2006). Work–home interaction as a framework specifies bidirectional (work-to-family and family-to-work) and either positive (enrichment) or negative (conflict) interactions between the work and home role of individuals (Geurts et al., 2005). Considering the scope of this paper, we focus on the positive work-to-family component (i.e., work–home enrichment). Work–home enrichment is a critical process through which resources developed at work (e.g., juggling multiple responsibilities) are transferred to the home role (e.g., better planning and prioritizing), resulting, as research demonstrates, in better functioning at work and home (Hakanen et al., 2011). Thus, we expect entrepreneurs who engage in proactive vitality management to transfer resources developed in their work role to potentiate their home role and thus amass further resources (spousal support; Powell and Eddleston, 2013). Creating such an extensive bundle of resources should allow entrepreneurs to handle their work and home roles efficiently. Perceiving that they thrive in both roles should generate increased entrepreneurial performance (Gorgievski et al., 2011; Wach et al., 2016). Figure 1 summarizes the proposed mediation model graphically.

#### Proactive Vitality Management and Work–Home Enrichment

Entrepreneurs who engage in proactive vitality management should be more likely to experience work–home enrichment. Our assumption is supported by one of the tenets of COR theory—those individuals who possess abundant resources find it easier to invest, attract, and gain additional resources (Hobfoll, 2011), and by empirical findings linking vitality to enrichment in the wage-employed sector (Bhave and Lefter, 2018). As presented, vital and energetic entrepreneurs at work should experience more positive interactions with various stakeholders (e.g., employees and clients), resulting in enhanced entrepreneurial performance (Zhou, 2015). However, this positive state does not vanquish instantly after leaving the office; based on the affective path of work–home enrichment (Greenhaus and Powell, 2006), entrepreneurs will transfer this positive state at home. The positive mood (e.g., positive affect) fostered in the work role will, through a spillover process, enable better interactions with spouses, family members, or friends (Bhave and Lefter, 2018).

Furthermore, an instrumental path of resource transfer is also to be expected. The instrumental path refers to the direct transference of resources, such as beneficial behaviors, from one role to another (Greenhaus and Powell, 2006). Striving to find inspiration, entrepreneurs could try a new café or bistro as a workspace for the day (proactive vitality management). Should they consider it a thrilling experience, they will also be more likely to visit the same place during non-working hours to socialize with actors from their private life, thus triggering an enrichment process. As such, entrepreneurs who employ proactive vitality management to optimize their functioning at work should experience work–home enrichment through the spillover of positive states (affective path) and behaviors (instrumental path) developed in their work role.

Also, we expect to find reciprocal effects between the two variables. Should entrepreneurs accumulate sufficient resources in their home role due to engaging in proactive vitality management, this should also create the premises for entrepreneurs to continue enacting resource investment behaviors and strategies. Building upon the previous example, spending a fun night out with loved ones in a newly discovered bistro can replenish one’s energy levels (Zijlstra and Cropley, 2006). Having their energy restored will then permit entrepreneurs to engage in further resource investment activities (e.g., preparing a nutritious breakfast) to optimize their work functioning. Considering these arguments, we state that:

*Hypothesis 2a:* Proactive vitality management at T1 has a positive impact on work–home enrichment at T2.

*Hypothesis 2b:* Work–home enrichment at T1 has a positive impact on proactive vitality management at T2.

*Hypothesis 2c:* Proactive vitality management and work–home enrichment have positive and reciprocal effects on each other over time.

#### Work–Home Enrichment and Entrepreneurial Performance

We also expect work–home enrichment to have a positive effect on entrepreneurial performance. Previous studies have demonstrated that work–home enrichment is positively related to job-related outcomes, including satisfaction and performance (for a meta-analysis, see Zhang et al., 2018). Individuals who employ resources developed at work (e.g., negotiating abilities) to enhance aspects of the home role (e.g., resolve conflicts) are more engaged and committed in their job, resulting in increased efficiency. However, only the reversed avenue—family-to-business enrichment—has been investigated in the entrepreneurship literature, with results capturing a cross-domain relationship between resources developed in the home role and enhanced business performance (Powell and Eddleston, 2013; Neneh, 2017). For instance, entrepreneurs can learn to be more caring in their role as spouses or parents and then transfer this ability to their work role when interacting with clients, resulting in increased performance.

Nevertheless, Crain and Hammer (2013) suggest that the work-to-family process usually occurs prior to family-to-work enrichment. The authors argue that individuals spend more time at work than home, especially in their young adulthood, and, as such, they first develop positive behaviors in their work role, which they then transfer to their home role, before learning to do the reverse (Crain and Hammer, 2013). Indeed, Zhang et al. (2018) also demonstrate that within-domain relationships (i.e., work–home enrichment and performance) are stronger than cross-domain relationships (family–work enrichment and performance), especially when it comes to enhancing performance. Furthermore, while Powell and Eddleston (2013) and Neneh (2017) find family-to-work enrichment to be a process that only occurs in the case of female entrepreneurs, Dinh et al. (2021) establish that the enrichment process functions for both male and female entrepreneurs. For this reason, we expect work–home enrichment to predict entrepreneurial performance, irrespective of gender.

To exemplify to proposed relationship, entrepreneurs who use organizing abilities developed at work to free up some time and spend a pleasant evening with family or friends will, through this action, accrue additional resources (i.e., spousal/social support; Powell and Eddleston, 2013) and have a feeling that their business and private life are balanced (Ezzedeen and Zikic, 2017). As such, they will be able, for instance, to negotiate home duties more efficiently and inhibit the onset of conflict in their home role due to not spending sufficient time with stakeholders from this role. Gleaning vital resources in the process will allow entrepreneurs to invest their conserved time and energy into another life domain they deem essential (Hobfoll, 2011), namely, developing their business. This should reflect in their entrepreneurial performance. Furthermore, perceiving how aspects pertaining to their work role enable them to handle their home role better should also allow entrepreneurs to have a more positive outlook on the future of their business (Wach et al., 2016).

Lastly, we expect to find reciprocal effects between work–home enrichment and entrepreneurial performance as well. Should entrepreneurs be satisfied with their business due to the transfer of resources from their work to their home role, this should also be reflected in a reversed relation. Entrepreneurs will foster additional resources (e.g., positive affect due to being satisfied with their firms’ profit) that will be transferred back to their home role, thus being able to handle the work–home mélange better. Based on these arguments, we posit that:

*Hypothesis 3a:* Work–home enrichment at T1 has a positive impact on entrepreneurial performance at T2.

*Hypothesis 3b:* Entrepreneurial performance at T1 has a positive impact on work–home enrichment at T2.

*Hypothesis 3c:* Work–home enrichment and entrepreneurial performance have positive and reciprocal effects on each other over time.

## Materials and Methods

### Participants & Procedure

Data were collected 1 month apart in January (Time 1; T1) and February 2021 (Time 2; T2), a time lag that permits the observation of changes in this studies’ variables (Dormann and Griffin, 2015; Bakker and van Wingerden, 2021). Participants had to be (1) founders and (2) owners of their firm to be considered entrepreneurs (Baron, 2007). Participants’ eligibility was verified by checking information from the founding and ownership statements of the entrepreneurs with the official records of the Romanian Ministry of Finance. The initial sample was reached using the snowball sampling technique and through recommendations from collaborators. The researchers contacted entrepreneurs *via* email, inviting them to participate in the study, and asked them to recommend other entrepreneurs who could be invited to participate. Before starting the survey, the entrepreneurs were informed about the aim of the study and assured about their data confidentiality. No incentives were offered for participation.

At T1, 277 Romanian entrepreneurs filled out the survey regarding the study variables. In the initial sample, respondents’ age ranged between 18–79 years old (*M* = 41.35, SD = 11.06), with the majority being male (60.3%). In terms of education, most respondents have at least a bachelor’s degree (65.7%), and the majority are either married (66.8%) or in a committed relationship (14.8%). The samples’ mean entrepreneurial experience is 12.12 years (SD = 12.05), and the mean firm tenure is 10.02 years (SD = 8.25). At T2, the same entrepreneurs were invited to fill out the survey again, resulting in 249 complete responses (cross-lagged response of 89.9%, relative to T1). The final sample has an age range between 20–80 years old (*M* = 41.88, SD = 11.19), again with most of the respondents being male (60.6%). The mean entrepreneurial experience is 12.13 years (SD = 9.11) and a mean business tenure of 9.85 years (SD = 8.29). The sample is heterogeneous in industries, spanning from consultancy services to construction, food industry, software development, or the hospitality industry.

### Instruments

We relied upon tried-and-tested instruments to measure this study’s variables. A Romanian version of the instruments was used, with all questionnaires having already been adapted (proactive vitality management; Bălăceanu et al., 2021) or used on Romanian samples in previous studies (work–home enrichment; Matei and Virga, 2020; entrepreneurial performance; Tisu and Vîrgă, 2021), where they yielded good psychometric properties.

Proactive vitality management was measured with the scale developed by Op den Kamp et al. (2018). The instrument has eight items, with answer options ranging on a Likert scale spanning from 1 (= totally disagree) to 7 (= totally agree). An example item is “I make sure that I feel energetic during my work.”

Work–home enrichment was assessed with a 5-item scale from the Survey Work-home Interaction—NijmeGen (SWING; Geurts et al., 2005). Answers were rated on a scale from 0 (= never) to 3 (= always). A sample item is: “You fulfill your domestic obligations better because of the things you have learned on your job?”

Entrepreneurial performance was measured with a set of five items from the scale developed by Stephan and Richter (2006). Responses were rated on 5-point Kunin faces scale, from 1 (= very dissatisfied) to 5 (= very satisfied). A sample item is: “How satisfied are you with your business income?”

### Data Analyses

#### Measurement Models and Measurement Invariance

To assess our measurement model, we conducted a confirmatory factor analysis (CFA) to verify the psychometric properties of our six-factor model (M6f; proactive vitality management, work–home enrichment, and entrepreneurial performance at T1 and T2). We conducted a longitudinal CFA to test for measurement invariance across the two measurement waves (Mackinnon et al., 2020). First, we tested a configural model (Mconfigural) in which we verified whether the hypothesized measurement model yields the same number of factors and configuration of item loadings across both waves. Then, we tested the metric invariance (Mmetric), where, building upon Mconfigural (nested), we also constrained all factor loadings to be equal across both waves. Next, building upon Mmetric, we tested for scalar invariance (Mscalar) by also constraining all intercepts to be equal across time. Finally, building upon Mscalar, we checked strict invariance (Mstrict), where, next to the constrained factor loadings and intercepts, we also constrained the residual errors to be equal across waves.

All procedures were carried out in R software (R Core Team, 2020), using the lavaan (Rossell, 2012) and semTools (Jorgensen et al., 2021) packages, based on maximum likelihood estimation. To assess model fit, we employed the following fit indices: the chi-square statistic (*χ*^2^), the comparative fit index (CFI), the Tucker–Lewis index (TLI), the root mean square error of approximation (RMSEA), and the standardized root mean square residual (SRMR). Following Marsh et al. (2005), we used the following cutoff points for acceptable fit: values of 0.90 or higher for CFI and TLI, and values equal or lower than 0.08 for RMSEA and SRMR. We inspected ∆CFI to assess differences between the measurement invariance models, with differences of 0.01 or lower indicating invariance (Chen, 2007; Mackinnon et al., 2020; Nawrocka et al., 2021).

#### Structural Models

The hypothesized models were tested *via* structural equation modeling (SEM) in R software (R Core Team, 2020) using the lavaan package (Rossell, 2012). We employed a full cross-lagged panel design, including proactive vitality management, work–home enrichment, and entrepreneurial performance at T1 and T2 as latent variables. Proactive vitality management and work–home enrichment were constructed using item-parceling, following the factorial algorithm procedure outlined by Rogers and Schmitt (2004). Thus, for proactive vitality management, we created three parcels consisting of 3 or 2 items, and for work–home enrichment, three parcels comprising 2 items each. This procedure was employed to obtain an adequate indicator-to-sample size ratio (Schreiber et al., 2006), given the difficulty in obtaining large samples of entrepreneurs for data collection (Taris et al., 2008).

To test the hypotheses, we used the analytical approach suggested by Cole and Maxwell (2003) and Taris and Kompier (2006), a procedure that allows testing partial mediation using a two-wave design (see also Hakanen et al., 2011; Nikolova et al., 2019; Nawrocka et al., 2021). To achieve our objective, we tested three cross-lagged models: (1) the potential causal relationship between the predictor (proactive vitality management) and the outcome (entrepreneurial performance), (2) the potential causal relationship between the predictor (proactive vitality management) and the mediator (work–home enrichment); and (3) the potential causal relationship between the mediator (work–home enrichment) and the outcome (entrepreneurial performance). In line with the recommended analyses, we tested four competing models: the stability model (Mstabil), where only autoregressive paths between the same set of variables are specified across time; the causality model (Mcausal) where, next to the autoregressive paths, a temporal causal relationship is introduced (for M1causal between proactive vitality management and entrepreneurial performance, for M2causal between proactive vitality management and work–home enrichment, and for M3causal between work–home enrichment and entrepreneurial performance); the reversed causation model (Mreversed), including autoregressive paths and the reversed hypothesized causal relationships (for M1reversed between entrepreneurial performance and proactive vitality management, for M2reversed between work–home enrichment and proactive vitality management, and for M3reversed between entrepreneurial performance and work–home enrichment); and the reciprocal model (Mreciprocal) including all the specified paths from Mstabil, Mcausal, and Mreversed together (in M1reciprocal we included M1stabil, M1causal and M1reversed, in M2reciprocal we included M2stabil, M2causal and M2reversed, and in M3reciprocal we included M3stabil, M3causal and M3reversed). Variables measured at the same time point were allowed to covary.

The same indicators (chi-square statistics, CFI, TLI, SRMR, and RMSEA) and cutoff values (0.90 or higher for CFI and TLI, 0.08 or lower for RMSEA and SRMR) as described in the model measurement section have been used to assess model fit. Model comparison of the structural models was carried out through a *χ*^2^ difference test.

## Results

### Attrition Analyses

Attrition analyses were conducted to test whether entrepreneurs who dropped out at T2 differ from entrepreneurs who completed both surveys regarding several demographic and this study’s variables (Bakker and van Wingerden, 2021). No statistically significant mean differences were found between the two groups.

### Descriptive Statistics and Correlations

Table 1 contains the means, standard deviations, and correlations between this study’s variables, as well as the internal consistency for each scale. All variables show positive, statistically significant relationships with each other, and the three constructs yield relatively high stability over time—proactive vitality management (*r* = 0.64, *p* < 0.001), work–home enrichment (*r* = 0.62, *p* < 0.001), and entrepreneurial performance (*r* = 0.76, *p* < 0.001). The internal consistency of the scales is good to excellent (0.85 lowest value—0.94 highest value; see Table 1).

**Table 1:** Means, standard deviations, correlation coefficients, and reliability coefficients table.

Variables	*M*	SD	1	2	3	4	5	6
*Time 1*								
1. Proactive vitality management_T1_	49.01	5.38	(0.90)					
2. Work–home enrichment_T1_	17.14	3.86	0.40^*^	(0.87)				
3. Entrepreneurial performance_T1_	18.42	3.81	0.27^*^	0.25^*^	(0.87)			
*Time 2*								
4. Proactive vitality management_T2_	47.42	5.94	0.64^*^	0.39^*^	0.34^*^	(0.94)		
5. Work–home enrichment_T2_	17.57	3.48	0.42^*^	0.62^*^	0.34^*^	0.47^*^	(0.85)	
6. Entrepreneurial performance_T2_	18.43	3.91	0.28^*^	0.35^*^	0.76^*^	0.41^*^	0.41^*^	(0.90)

NT_1_ = 277; NT_2_ = 249.

* *p < 0.001*.

*Cronbach’s α coefficients are displayed on the main diagonal*.

### Measurement Models and Measurement Invariance

Results of the conducted CFA are reported in Table 2. Upon initial inspection of our six-factor measurement model, we noticed that one item from the entrepreneurial performance scale (i.e., “How satisfied are you with the reputation of your firm?”) had a factor loading below 0.40 at both measurement times. The poor loading of this item is probably tied to the poor fit indices for the entire measurement model (M6f). Considering this, we decided to eliminate this item from the scale (Hakanen et al., 2011) and test an improved six-factor model (M6fimp)., The improved six-factor model (M6fimp) yields satisfactory fit indices *χ*^2^(579) = 1230.97, *p* < 0.001, CFI = 0.90; TLI = 0.89; RMSEA = 0.06, 90% CI [0.06–0.07], SRMR = 0.06. Thus, we proceeded to test measurement invariance and structural models using the improved six-factor model.

**Table 2:** Fit statistics for the measurement models and measurement invariance.

Model	Model description	*χ^2^*	*df*	CFI	TLI	RMSEA [90% CI]	SRMR	Model comparisons	*Δχ^2^*	*Δdf*	*Δ*CFI
*Measurement models*											
M_6f_	Measurement model—6 factors	1346.15	613	0.88	0.87	0.07 [0.06–0.07]	0.06				
M_6fimp_	Improved measurement model—6 factors (one EP item removed)	1230.97^*^	579	0.90	0.89	0.06 [0.06–0.07]	0.06				
*Measurement invariance*											
M_configural_	Model for configural invariance	1050.26^*^	569	0.93	0.92	0.06 [0.05–0.06]	0.06				
M_metric_	Model for metric invariance (constrained all FL to be equal)	1072.97^*^	587	0.92	0.92	0.06 [0.05–0.06]	0.07	M_configural_ vs. M_metric_	20.03	18	0.003
M_scalar_	Model for scalar invariance (constrained all FL and I to be equal)	1162.77^*^	605	0.91	0.91	0.06 [0.05–0.06]	0.07	M_metric_ vs. M_scalar_	92.67^*^	18	0.007
M_strict_	Model for strict invariance (constrained all FL, I, and RE to be equal)	1313.35^*^	623	0.90	0.89	0.06 [0.05–0.07]	0.07	M_scalar_ vs. M_strict_	70.97^*^	18	0.010

*N(T1) = 277; N(T2) = 249*.

* *p < 0.001*.

*EP*, *Entrepreneurial performance; FL, Factor loadings; I, Intercepts; RE, Residual errors.*

All measurement invariance models—Mconfigural, Mmetric, Mscalar, and Mstrict yield satisfactory fit indices regarding the measurement invariance across the two waves. The best fit was shown by the configural model, with the chi-square difference tests indicating a decrease in model fit for the other models, hinting at the possibility that the data were not invariant. However, Chen (2007) argues that the chi-square difference test is too sensitive to test measurement invariance, suggesting an inspection of the CFI difference between models as a more robust alternative (see also (Mackinnon et al., 2020; Nawrocka et al., 2021). Upon inspecting ∆CFI, the difference between the configural and metric model, between the metric and scalar model, and between the scalar and strict model was equal or less than 0.01, indicating measurement invariance. There are slim chances that the variance in the structural models was obtained due to measurement fluctuations, and thus we proceeded to test the structural models.

### Hypothesized Cross-Lagged Structural Models

**Figure 2:**
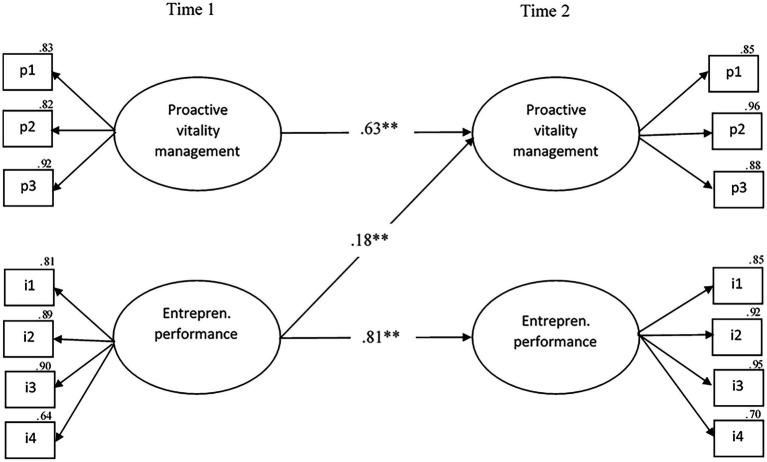
The final model of the statistically significant cross-lagged relationships between proactive vitality management and entrepreneurial performance. *N* = 249, ^*^p < 0.01, ^**^p < 0.001, pl−p3 parcels 1 through 3, i1–i4 = items 1 through 4. entrepren = entrepreneurial.

Tables 3–5 show the results for the mediation model using structural cross-lagged models. Regarding the potential causal relationship between proactive vitality management (predictor) and entrepreneurial performance (outcome), all structural models yield acceptable fit indices (see Table 3). The reversed model (M1reversed) is statistically superior to the stability model (M1stabil; Δ*χ*^2^(2) = 10.82, *p* < 0.01). The reciprocal model (M1reciproc) is also superior to the stability model (M1stabil; Δ*χ*^2^(2) = 12.77, *p* < 0.01) and the causal model (M1causal; Δ*χ*^2^(2) = 10.26, *p* < 0.01), but not the reciprocal model (M1reversed; Δ*χ*^2^(1) = 1.95, *p* > 0.05). Both in the causal and the reciprocal model the relationship between proactive vitality management at T1 and entrepreneurial performance at T2 (H1a) was non-significant (M1causal; *β* = 0.08, *p* = 0.12; M1reciproc; *β* = 0.06, *p* = 0.16). Thus, the reversed model (M1reversed) is the best model where all relationships are statistically significant, leading us to consider the reversed model as the single acceptable model (M1reversed; *χ*^2^(72) = 197.12, *p* < 0.001, CFI = 0.96; TLI = 0.95; RMSEA = 0.08, 90% CI [0.07–0.10], SRMR = 0.05). As depicted in Figure 2, in M1reversed, proactive vitality management at T1 predicts itself at T2 (*β* = 0.63, *p* < 0.001), while entrepreneurial performance at T1 predicts itself at T2 (*β* = 0.83, *p* < 0.001) as well as proactive vitality management at T2 (H1b; *β* = 0.18, *p* = 0.001).

**Table 3:** Fit statistics for the cross-lagged structural models between proactive vitality management and entrepreneurial performance.

Model	Model description	*χ^2^*	*Df*	CFI	TLI	RMSEA [90% CI]	SRMR	Model comparisons	*Δχ^2^*	*Δdf*
M1_stabil_	Stability model	207.94^**^	73	0.95	0.94	0.08 [0.07–0.10]	0.08			
M1_causal_	Causal model (M1_stabil_ + PVM→EP)	205.43^**^	72	0.95	0.94	0.08 [0.07–0.10]	0.07	M1_stabil_ vs. M1_causal_	2.51	1
								M1_stabil_ vs. M1_reversed_	10.82^*^	1
M1_reversed_	Reversed model (M1_stabil_ + EP→PVM)	197.12^**^	72	0.96	0.95	0.08 [0.07–0.10]	0.05	M1_stabil_ vs. M1_reciproc_	12.77^*^	2
								M1_causal_ vs. M1_reciproc_	10.26^*^	1
M1_reciproc_	Reciprocal model (M1_causal_ + M1_reversed_)	195.16^**^	71	0.96	0.95	0.08 [0.07–0.10]	0.05	M1_reversed_ vs. M1_reciproc_	1.95	1

*N = 249*.

* *p < 0.01*.

** *p < 0.001*.

*PVM, Proactive vitality management, EP, Entrepreneurial performance*

**Table 4:** Fit statistics for the cross-lagged structural models between proactive vitality management and work–home enrichment.

Model	Model description	*χ^2^*	*Df*	CFI	TLI	RMSEA [90% CI]	SRMR	Model comparisons	*Δχ^2^*	*Δdf*
M2_reciproc_	Stability model	99.94^**^	50	0.98	0.97	0.06 [0.05–0.08]	0.07			
M2_causal_	Causal model (M2_stabil_ + PVM→WHE)	90.54^**^	49	0.98	0.97	0.06 [0.04–0.08]	0.04	M2_stabil_ vs. M2_causal_	9.41^*^	1
								M2_stabil_ vs. M2_reversed_	8.46^*^	1
M2_reversed_	Reversed model (M2_stabil_ + WHE→PVM)	91.48^**^	49	0.98	0.97	0.06 [0.04–0.08]	0.05	M2_stabil_ vs. M2_reciproc_	16.36^**^	2
								M2_causal_ vs. M2_reciproc_	6.95^*^	1
M2_reciproc_	Reciprocal model (M2_causal_ + M2_reversed_)	83.59^**^	48	0.98	0.98	0.06 [0.04–0.07]	0.03	M2_reversed_ vs. M2_reciproc_	7.89^*^	1

*N = 249*.

* *p < 0.01*;

** *p < 0.001*.

*PVM, Proactive vitality management, WHE, Work–home enrichment.*

**Table 5:** Fit statistics for the cross-lagged structural models between work–home enrichment and entrepreneurial performance.

Model	Model description	*χ2*	*Df*	CFI	TLI	RMSEA [90% CI]	SRMR	Model comparisons	*Δχ2*	*Δdf*
M3_stabil_	Stability model	169.24^**^	73	0.96	0.95	0.07 [0.06–0.09]	0.07			
M3_causal_	Causal model (M3_stabil_ + WHE→EP)	155.99^**^	72	0.97	0.96	0.07 [0.05–0.08]	0.06	M3_stabil_ vs. M3_causal_	13.25^**^	1
								M3_stabil_ vs. M3_reversed_	10.05^*^	1
M3_reversed_	Reversed model (M3_stabil_ + EP→WHE)	159.19^**^	72	0.97	0.96	0.07 [0.06–0.08]	0.05	M3_stabil_ vs. M3_reciproc_	22.63^**^	2
								M3_causal_ vs. M3_reciproc_	9.38^*^	1
M3_reciproc_	Reciprocal model (M3_causal_ + M3_reversed_)	146.61^**^	71	0.97	0.96	0.07 [0.05–0.08]	0.04	M3_reversed_ vs. M3_reciproc_	12.58^**^	1

*N = 249*.

* *p < 0.01*;

** *p < 0.001*.

*WHE, Work–home enrichment, EP, Entrepreneurial performance.*

**Figure 3:**
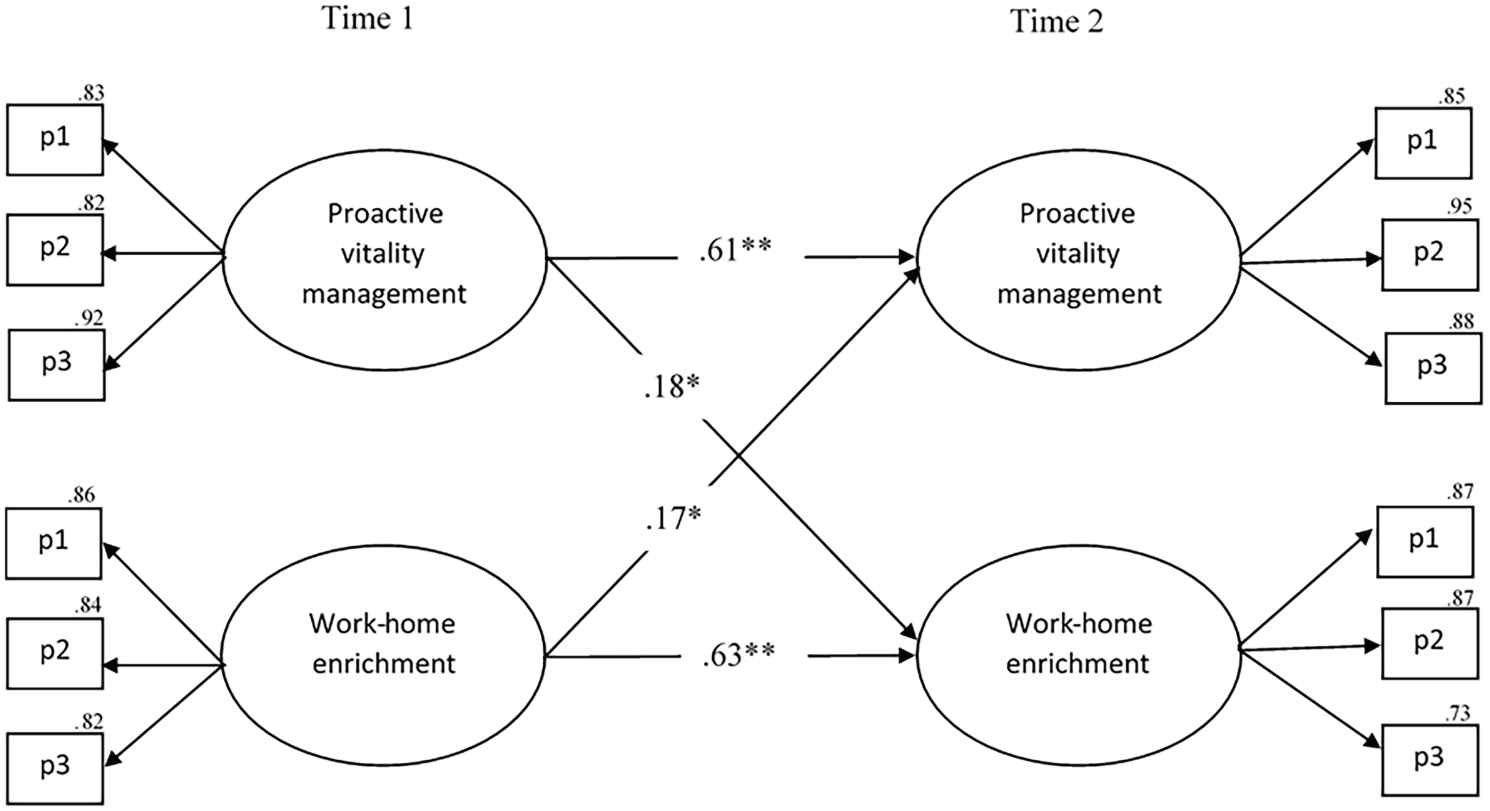
The final model of the statistically significant cross-lagged relationships between proactive vitality management and work–home enrichment. *N* = 249, ^*^p < 0.01, ^**^p < 0.001. P1−P3 = parcels 1 through 3.

Regarding the potential causal relationship between proactive vitality management (predictor) and work–home enrichment (mediator), all structural models yield excellent fit indices (see Table 4). The best-fitting model is the reciprocal model (M2reciprocal; *χ*^2^(48) = 83.59, *p* < 0.001, CFI = 0.98; TLI = 0.98; RMSEA = 0.06, 90% CI [0.04–0.07], SRMR = 0.03), which is statistically significantly better than the stability model (M2stabil; Δ*χ*^2^(2) = 16.36, *p* < 0.001), the causal model (M2causal; Δ*χ*^2^(1) = 6.95, *p* < 0.01), and the reversed model (M2reversed; Δ*χ*^2^(1) = 7.89, *p* < 0.01). This suggests that there is both a potential causal and a reversed relationship between the predictor and mediator. As depicted in Figure 3, all relationships between proactive vitality management and work–home enrichment are positive and statistically significant in the M2reciprocal model. Specifically, proactive vitality management at T1 predicts proactive vitality management at T2 (*β* = 0.61, *p* < 0.001) and work–home enrichment at T2 (H2a; *β* = 0.18, *p* < 0.01). Conversely, work–home enrichment at T1 predicts itself at T2 (*β* = 0.63, *p* < 0.001), and proactive vitality management at T2 (H2b; *β* = 0.17, *p* < 0.01).

**Figure 4:**
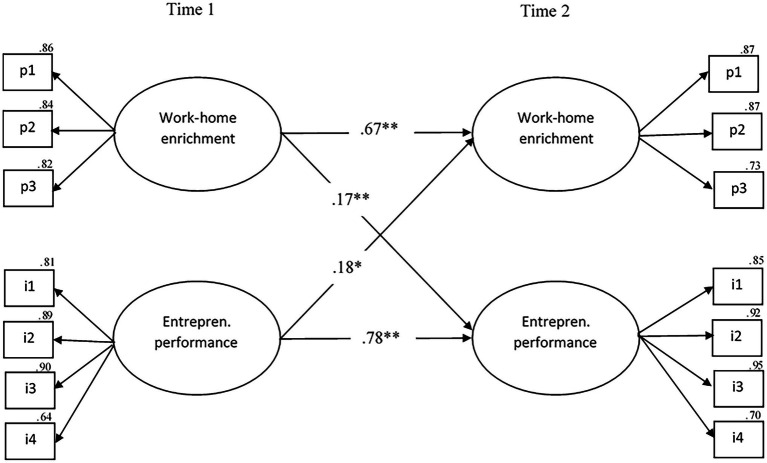
The final model of the statistically significant cross-lagged relationships between work–home enrichment and entrepreneurial performance. *N* = 249, ^*^p < 0.01, ^**^p < 0.001, pl−p3 parcels 1 through 3, i1–i4 = items 1 through 4. entrepren = entrepreneurial.

Regarding the potential causal relationship between work–home enrichment (mediator) and entrepreneurial performance (outcome), all structural models also yield excellent fit indices (see Table 5). The best-fitting model is, again, the reciprocal model (M3reciprocal; *χ*^2^(71) = 146.61, *p* < 0.001, CFI = 0.97; TLI = 0.96; RMSEA = 0.07, 90% CI [0.05–0.08], SRMR = 0.04), which is statistically significantly better than the stability model (M3stabil; Δ*χ*^2^(2) = 22.63, *p* < 0.001), the causal model (M3causal; Δ*χ*^2^(1) = 9.38, *p* < 0.01), and the reversed model (M3reversed; Δ*χ*^2^(1) = 12.58, *p* < 0.001). This suggests that there is both a potential causal and a reversed relationship between the mediator and outcome. As shown in Figure 4, all relationships between work–home enrichment and entrepreneurial performance are positive and statistically significant in the M3reciprocal model. Specifically, work–home enrichment at T1 predicts work–home enrichment at T2 (*β* = 0.66, *p* < 0.001) and entrepreneurial performance at T2 (H3a; *β* = 0.14, *p* < 0.01). Conversely, entrepreneurial performance at T1 predicts itself at T2 (*β* = 0.80, *p* < 0.001), and work–home enrichment at T2 (H3b; *β* = 0.17, *p* < 0.01).

Summarizing, the hypothesized mediation model was partly confirmed. Proactive vitality at T1 did not predict entrepreneurial performance at T2 thus rejecting Hypothesis 1a, while entrepreneurial performance at T1 was a predictor of proactive vitality management at T2 conferring support to Hypothesis 1b. Furthermore, proactive vitality management at T1 did predict work–home enrichment at T2, and work–home enrichment at T1 was an antecedent of proactive vitality management at T2, conferring support to Hypotheses 2a and 2b. Hypotheses 3a and 3b also found support, with work–home enrichment at T1 being a precursor of entrepreneurial performance at T2 and entrepreneurial performance at T1 predicting work–home enrichment at T2. Regarding the proposed mutual effects between the variables, Hypothesis 1c was partly supported, because only entrepreneurial performance had a cross-lagged effect on proactive vitality management (M1reversed). Additionally, we established that proactive vitality management and work–home enrichment have a positive and reciprocal effect on each other over time (M2reciprocal), favoring Hypothesis 2c. That was also the case in the relationship between work–home enrichment and entrepreneurial performance, which show a positive and reciprocal effect on each other over time (M3reciprocal), providing support to Hypothesis 3c. Thus, work–home enrichment appears to be a mediator in the linkage between entrepreneurs’ proactive vitality management and entrepreneurial performance.

## Discussion

Rooted in the JD-R (Bakker and Demerouti, 2017) and COR (Hobfoll, 2011) theories, the current investigation proposed a model where proactive vitality management (a behavioral component) is an antecedent of entrepreneurial performance (work role), employing work–home enrichment (home role) as an explanatory mechanism. The investigated mediation model was tested by means of a two-wave cross-lagged model. We assumed both potential temporal causal relationships between the variables and reciprocal effects. Results partially supported our assumptions. Most pairs of variables (i.e., proactive vitality management and work–home enrichment, and work–home enrichment and entrepreneurial performance), show mutual effects on each other over time, hinting at a mediation mechanism. Proactive vitality management, however, is only a distal precursor of entrepreneurial performance. Nevertheless, results suggest the work and home role of entrepreneurs can, indeed, be allies, exhibiting positive reciprocal effects through a transfer of resources from one role to potentiate aspects of the other.

Contrary to our expectations, in the relationship between predictor and outcome variable, the reversed model is the best-fitting model. Entrepreneurial performance has a direct effect on entrepreneurs engaging in proactive behaviors directed at optimizing their energy levels for work, but not the other way around. Based on our results, self-regulatory actions, such as exercising in the morning or meditating about the meaning of their job, do not have a direct effect on entrepreneurs’ satisfaction with aspects of their business. While previous studies establish a correlational link between proactive vitality management and performance (Op den Kamp et al., 2018; Ye et al., 2020), predictive analyses employed in this study show that this linkage is mediated by different mechanisms (e.g., work–home enrichment). This result aligns with the proposition of the JD-R theory, which places mediating variables (i.e., work engagement) as explanatory links in the relationship between individual strategies and performance outcomes (Bakker and Demerouti, 2017). However, those entrepreneurs who are more satisfied with their income or number of employees do employ strategies directed at managing their physical and mental energies to promote functioning at work. COR theory provides theoretical coverage for this result. Individuals who possess sufficient resources (e.g., entrepreneurial performance) are more inclined to invest existing resources (e.g., time and energy) for further resource gains (e.g., proactive vitality management), probably due to existing autonomous motivation (Stephan et al., 2020).

COR theory can also best explain the mutual effects between predictor and mediator, and between mediator and outcome captured in this study. First, engaging in proactive vitality management appears to trigger a resource gain cycle, where entrepreneurs transfer resources between their work role and their home role. For instance, entrepreneurs can meditate about the meaning of their work and how their business helps their community (proactive vitality management). This can generate a positive state (Zeng et al., 2015) which can be transferred to their home role through the affective path proposed by Greenhaus and Powell (2006), where they will be more receptive to domestic needs (work–home enrichment). Conversely, when entrepreneurs experience less conflict in their home role (Ezzedeen and Zikic, 2017) and more support from their spouse (Powell and Eddleston, 2013), especially due to abilities developed in their work role (e.g., conflict resolution skills), they will possess sufficient resources to also engage in further proactive behaviors that can boost their energy levels, such as playing an instrument for inspiration or jogging in the morning (proactive vitality management). Our results are in line with the findings of Bhave and Lefter (2018), who also found vitality to be an antecedent of work–home enrichment and the tenets of COR theory. Those entrepreneurs who possess an abundance of resources are more prone to reinvest them for further resource gain (Hobfoll, 2011).

Similarly, when entrepreneurs experience work–home enrichment, thus being satisfied with how aspects of their work enhance their home functioning, they also appear to be more satisfied with their entrepreneurial performance. Our results complement and extend the findings of Zhang et al. (2018), who establish that work–home enrichment is a precursor of employee satisfaction and performance. When individuals perceive that their work role potentiates their home role, they appear to become more dedicated and productive at work (Crain and Hammer, 2013; Zhang et al., 2018), with entrepreneurs making no exception. Based on our results, entrepreneurs who, for instance, develop planning and prioritizing skills at work due to a packed schedule can employ these to thrive in their home role (e.g., better scheduling of family duties). Considering that entrepreneurs deem their personal life as a vital element linked to their business success (Walker and Brown, 2004; Kirkwood, 2016), the above enrichment process is bound to reflect in a more positive evaluation of their firm performance. Furthermore, entrepreneurial performance allows entrepreneurs to direct additional resources to their home role (e.g., spending more time with family or friends), potentiating the work–home enrichment process. Specifically, entrepreneurs who report high entrepreneurial performance will be prone to invest current resources (e.g., time and energy) in life domains other than their business (Ezzedeen and Zikic, 2017), thus enabling the occurrence of work–home enrichment, aspect that aligns with the propositions of COR theory (Hobfoll, 2011).

Summarizing, our results highlight that the work and home role of entrepreneurs can, in fact, act as potential allies, yet entrepreneurs must develop and employ relevant behavioral tools (e.g., proactive vitality management) to handle both roles efficiently. Furthermore, the model underlines the importance of integrating the role of an individual with a private life into models investigating factors related to entrepreneurs’ success indicators. Examining both sides of the coin (i.e., work and home role) in a dynamic, longitudinal manner will enable more accurate modeling of factors that shape entrepreneurial performance.

### Limitations and Future Directions

Some limitations of the current study are to be noted. First, we relied on self-report questionnaires to obtain the data and assess this studies’ variables. While this is a common practice in psychological studies, especially in ones that evaluate participants’ perceptions (Conway and Lance, 2010), it also makes the data susceptible to common method bias (CMB; Podsakoff et al., 2012). The time-lagged nature of the study corroborated with the good fit indices of our measurement and invariance models suggests a slim chance for the occurrence of CMB. However, future studies could use more diverse (e.g., peer-ratings) and objective (e.g., time spent with family or friends) instruments to capture this study’s variables. It should also be noted that, in this study, the outcome variable referred to entrepreneurs’ perceptions about the performance of their business. Therefore, future studies should aim to replicate our findings in relation to more objective indicators of business performance (e.g., actual income or profit).

Second, while it has been argued that cross-lagged studies enable researchers to capture causal relationships among variables (Little, 2013; Nikolova et al., 2019), other scholars argue that such an approach only hints at potential causal, temporal relationships that need to be tested using experimental designs to infer causal conclusions (see Bradford Hill criteria; Hamaker et al., 2015; Cox, 2018). As such, while our longitudinal approach permits assumptions of causality between proactive vitality management, work–home enrichment, and entrepreneurial performance, future studies should test our model by means of randomized controlled trials to test whether modifications in this study’s variables determine an enhancement in entrepreneurial performance. Considering that existing interventions aimed at developing one’s vitality (Hendriksen et al., 2016) or work–home enrichment (Heskiau and McCarthy, 2020) have been linked to an increase in positive work-related outcomes, we expect our model to also pass the scrutiny of an experimental trial.

Third, our sample consists of entrepreneurs from Eastern Europe, which may inhibit the generalization of our findings. While some authors argue that entrepreneurs are similar in characteristics irrespective of their cultural background (Turan and Kara, 2007), contextual factors, such as economic, institutional, or cultural variables, appear to shape entrepreneurial outcomes (Fernández-Serrano et al., 2018; Nikolaev et al., 2018). As such, future studies should aim to replicate our findings on other cultures with different cultural and/or economic backgrounds. Furthermore, we collected data at only two time points; to capture potential full mediation models, a minimum of three waves would have been necessary (Hakanen et al., 2011; Nikolova et al., 2019).

### Theoretical and Practical Implications

This study contributes to entrepreneurship literature in several important ways. First, heeding the call of Kleine and Schmitt (2021), who encourage researchers to uncover malleable behavioral mechanisms related to entrepreneurial wellbeing and success, we identify proactive vitality management as a vital antecedent linked to entrepreneurs thriving in their business role as well as in their personal life. As such, we demonstrate that entrepreneurs ought to engage in behaviors aimed at managing their physical and mental energies to promote their work because this is beneficial in juggling work and domestic responsibilities efficiently. Proactive vitality management appears to trigger a resource-gaining cycle (Hobfoll, 2011), fueling entrepreneurs’ work–home enrichment, which, in turn, provides the necessary resources for entrepreneurs to increase their entrepreneurial performance. Furthermore, both enrichment and performance will then generate the premises for further engagement in individual strategies. Thus, the proposed individual strategy helps entrepreneurs foster positive, proactive behaviors to enhance functioning in their life, satisfying a central tenet of the positive psychology framework (Seligman and Csikszentmihalyi, 2014).

Second, by identifying proactive vitality management as a precursor of entrepreneurial success, our study also demonstrates that the recent JD-R expansion—the inclusion of individual strategies into the theoretical framework (Bakker and Demerouti, 2017; Demerouti et al., 2019), applies to the entrepreneurship literature as well. Engaging in self-regulatory actions to increase existing resources and avoid loss, such as proactive vitality management, is beneficial for entrepreneurs, leading to various positive outcomes. However, it can be argued that proactive vitality management occurs mostly outside the office, with entrepreneurs employing other types of strategies once at the office. This could be a potential explanation why we found no direct longitudinal link between proactive vitality management and entrepreneurial performance. Indeed, proactive vitality management is one individual strategy among many (i.e., strengths use, job crafting; Demerouti et al., 2019). Therefore, we second the call of Kleine and Schmitt (2021) and encourage researchers to incorporate other such individual strategies into models investigating entrepreneurs’ success determinants both in and outside of work. Tisu and Vîrgă (2021), for instance, have already established that strengths use allows entrepreneurs to capitalize on existing growth opportunities, indicating that this is a ripe area of investigation. Furthermore, to fully capture the essence of the JD-R theory in the entrepreneurship literature, scholars should also seek to integrate personal resources into research models. As research demonstrates, beliefs about being able to control their environment (i.e., personal resources; Bakker and Demerouti, 2017) can mitigate the impact of stressors on strain (Arshi et al., 2020), as well as potentiate the positive effect of individual strategies (Demerouti et al., 2019).

Third, we managed to establish a longitudinal causal relationship between entrepreneurs’ work and home roles from a positive perspective. This is a relatively new and unexplored area in entrepreneurship research that requires the focus of scholars to better understand the interplay between the two roles entrepreneurs assume. Although scholars tend to agree that the work and home roles of entrepreneurs are closely intertwined (Stephan, 2018), they acknowledge that longitudinal investigations are warranted to assess potential causal relationships between entrepreneurs work and home roles (Powell and Eddleston, 2013; Ezzedeen and Zikic, 2017). This study addresses this issue. By identifying positive dynamic relationships between proactive vitality management, work–home enrichment, and entrepreneurial performance, we demonstrate that the two roles entrepreneurs assume ought not always be in conflict (Arshi et al., 2020, 2021). They can also act as potential allies, results that align with the propositions of the positive psychology framework. Individuals are happier when they successfully integrate multiple roles efficiently, thriving in them concomitantly (Seligman and Csikszentmihalyi, 2014). Specifically, the mutual positive effects between aspects of the work role (i.e., entrepreneurial performance) and home role (i.e., work–home enrichment) highlight the fact that entrepreneurs should avoid sacrificing one role for another (Ezzedeen and Zikic, 2017; Adisa et al., 2019), and employ strategies that enable them to bloom in both. This will be beneficial for entrepreneurs’ overall wellbeing (Stephan, 2018) and thus probably secure the long-term survival and growth of the business (Wach et al., 2016).

From a practical perspective, we provide entrepreneurs and practitioners with a set of moldable tools that can be cultivated to allow entrepreneurs to manage their work and home role efficiently and concomitantly. Regarding the development of vitality management, scholars indicate that individuals can engage in various activities, such as practicing sports in the morning (e.g., jogging), keeping a balanced diet, or ensuring they get enough sleep at night (Hendriksen et al., 2016; Schmitt et al., 2017). Importantly, proactive vitality management is idiosyncratic. Entrepreneurs ought to test various such practices and retain and employ those activities that they feel best suit their momentary needs.

Additionally, Heskiau and McCarthy (2020) also suggest a series of activities that can help individuals foster work–home enrichment. Entrepreneurs can seek to actively acknowledge work resources that can be transferred to the home role (e.g., computer skills). They can also generate positive connections across roles or engage in mental practices, such as visualizing the steps to success, that can increase confidence in transferring resources across roles (Heskiau and McCarthy, 2020). Taken together, these activities are bound to reinforce each other and should help entrepreneurs secure increased entrepreneurial performance.

## Conclusion

Entrepreneurs’ work and private life are closely intertwined, yet entrepreneurs can find it challenging to juggle between the two competing roles and satisfy both. This study uncovered proactive vitality management as a malleable behavioral component that helps entrepreneurs handle their work and home role efficiently, by transferring resources from their work role to enhance aspects of their home role. As such, entrepreneurs will ensure that work-related activities can help satisfy both their social (work–home enrichment) and financial (entrepreneurial performance) aspirations, reflecting on their entrepreneurial performance. Results also reveal that the two roles entrepreneurs assume have mutually beneficial effects on each other over time. When their work and home role act as allies, this enables entrepreneurs to enter a positive resource gain cycle. One concrete strategy toward attaining this objective is to engage in behaviors directed at managing mental and physical energies to promote optimal functioning at work.

## Data Availability Statement

The raw data supporting the conclusions of this article will be made available by the authors, without undue reservation.

## Ethics Statement

Ethical review and approval was not required for the study on human participants in accordance with the local legislation and institutional requirements. The patients/participants provided their written informed consent to participate in this study.

## Author Contributions

LT and DV conceived the presented idea. LT developed the theory and performed the computations. DV verified the analytical methods and guided the theoretical argumentation process. All authors contributed to the article and approved the submitted version.

## Conflict of Interest

The authors declare that the research was conducted in the absence of any commercial or financial relationships that could be construed as a potential conflict of interest.

## Publisher’s Note

All claims expressed in this article are solely those of the authors and do not necessarily represent those of their affiliated organizations, or those of the publisher, the editors and the reviewers. Any product that may be evaluated in this article, or claim that may be made by its manufacturer, is not guaranteed or endorsed by the publisher.
